# Enhancing pre-clinical research with simplified intestinal cell line models

**DOI:** 10.1177/20417314241228949

**Published:** 2024-03-04

**Authors:** Christina Fey, Theresa Truschel, Kristina Nehlsen, Spyridon Damigos, Julia Horstmann, Theresia Stradal, Tobias May, Marco Metzger, Daniela Zdzieblo

**Affiliations:** 1 Translational Center for Regenerative Therapies (TLZ-RT) Würzburg, Branch of the Fraunhofer Institute for Silicate Research (ISC), Würzburg, Germany; 2 InSCREENeX GmbH, Braunschweig, Germany; 3 Department of Tissue Engineering and Regenerative Medicine (TERM), University Hospital Würzburg, Würzburg, Germany; 4 Helmholtz Centre for Infection Research, Braunschweig, Germany; 5 Project Center for Stem Cell Process Engineering (PZ-SPT), Branch of the Fraunhofer Institute for Silicate Research (ISC), Würzburg, Germany

**Keywords:** Small intestinal tissue, immortalization, organoids, cell line, Transwell® models

## Abstract

Two-dimensional culture remains widely employed to determine the bioavailability of orally delivered drugs. To gain more knowledge about drug uptake mechanisms and risk assessment for the patient after oral drug admission, intestinal in vitro models demonstrating a closer similarity to the in vivo situation are needed. In particular, Caco-2 cell-based Transwell® models show advantages as they are reproducible, cost-efficient, and standardized. However, cellular complexity is impaired and cell function is strongly modified as important transporters in the apical membrane are missing. To overcome these limitations, primary organoid-based human small intestinal tissue models were developed recently but the application of these cultures in pre-clinical research still represents an enormous challenge, as culture setup is complex as well as time- and cost-intensive. To overcome these hurdles, we demonstrate the establishment of primary organoid-derived intestinal cell lines by immortalization. Besides exhibiting cellular diversity of the organoid, these immortalized cell lines enable a standardized and more cost-efficient culture. Further, our cell line-based Transwell®-like models display an organ-specific epithelial barrier integrity, ultrastructural features and representative transport functions. Altogether, our novel model systems are cost-efficient with close similarity to the in vivo situation, therefore favoring their use in bioavailability studies in the context of pre-clinical screenings.

## Introduction

The intestinal tract plays a crucial role in the digestion of food as well as the absorption of water and important nutrients.^1–3^ In addition, the intestinal epithelium forms a tight barrier with the overlying intestinal mucosa that presents pathogenic microorganisms or other harmful substances from entering the bloodstream.^4,5^

Intestinal epithelial cells (IECs) represented by enterocytes, goblet cells, enteroendocrine cells, Paneth cells, stem cells, and some rare cell types^6–8^ play a key role for nutrient digestion, absorption, barrier integrity, and intestinal homeostasis. Mimicking the cellular composition and versatile functions in vitro would lead to predictive as well as representative in vitro models, thereby reducing animal studies and failure rates in clinical trials of newly developed drugs. Therefore, cell-based models are routinely used in fundamental, translational, or preclinical research applications with distinct complexities ranging from simple two-dimensional (2D) cell culture models to more complex multicellular three-dimensional (3D) models to recreate the in vivo structural and functional organization. However, one of the major challenges in mimicking the native intestinal epithelium in vitro remains the selection of an application-specific cell source and the resulting appropriate cell composition.

The most widely used cell sources are human adenocarcinoma-derived cell lines such as Caco-2 or HT-29.^9,10^ Tumor-derived cell lines cultured on collagen-coated semi-permeable polyethylene terephthalate (PET)-based Transwell®-inserts display in vivo-like characteristics to some extent, like the formation of a polarized monolayer, the expression of specialized transporters (e.g. Mdr1/GLUT1) in the apical brush border, the development of differentiated cells resembling for example, mature enterocytes or other specified intestinal cell types as well as the formation of cell-cell interactions between neighboring cells. Further, these models are easy to handle under cost-efficient conditions and enable drug absorption studies in a high-throughput screening (HTS) approach.^10^ Despite many advantages, however, these models demonstrate limitations with regard to recreating a physical barrier induced by mucus-secreting goblet cells^11^ or an adequate expression of tight junction proteins, drug transporters, or metabolic enzymes, resulting in a lower predictivity in drug screenings.^12,13^

Currently, human primary organoid cultures are regarded as advanced model systems, as these organotypic 3D-structures reflect the in vivo situation to a high degree by forming representative tight junctions and developing a crypt-villus axis with a similar organization of the different characteristic cell types (enterocytes, goblet cells, enteroendocrine cells, Paneth cells, and stem cells) to that found in the small intestinal epithelium. Additionally, methods for generating polarized monolayers of these organoid cultures were established to improve the versatility of these models, for example, to study drug transport or to investigate cellular communication with other cell types located in the underlying lamina propria.^14–16^ Such models depend on culture conditions regulating intestinal stem cell (ISC) proliferation followed by inducing differentiation of ISCs toward the above-mentioned cell types. The differentiation process is controlled to some degree by varying of growth factors such as Wnt3a, SB202190, or Nicotinamide supplemented into the culture medium.^14,17^ However, there are some unresolved issues that need to be addressed to ensure reproducible results and full recapitulation of the in vivo physiology. Organoids can also differ in their cell type composition and show limitations in their availability with a specific genetic background. Furthermore, 3D culture conditions and supplementation of versatile growth factors require time- and cost-intensive handling in the lab, which supports the idea to use alternative cell sources to model the small intestine in vitro.

In view of the positive features of primary organoid cultures, we hypothesized that primary cell-derived, immortalized intestinal cell lines would combine a standardized and cost-efficient culture together with the preservation of the cellular subtypes localized in the native intestinal epithelium. The present study describes the generation and characterization of a stable and polarized cell line after lentiviral transduction with a defined set of immortalization genes. The established cell lines are cultured and expanded in 2D, thereby enabling easy handling under cost-efficient conditions. Both functional and structural features of the established cell lines of murine and human origin are demonstrated by immunofluorescence analysis, electron microscopy, barrier integrity measurements, and transport studies with representative reference substances. Taken together, the established murine and human cell lines represent alternative cell sources for the setup of a more standardized, cost-efficient intestinal in vitro model with improved barrier characteristics for bioavailability or toxicity studies in pre-clinical research applications.

## Materials and methods

### Animal handling

Animal research was performed according to German law and institutional guidelines approved by the institutional board of animal protection. Animals (C57BL/6 mice) used for small intestinal crypt isolation received proper attention and human care in compliance with the Guide for Care and Use of Laboratory Animals published by the National Institute of Health (NIH publication no.85e23, revised 1996). Mice were housed with free access to food and water *ad libitum* at 12 h light and dark cycles and were fed with a standard chow obtained from Altromin (Lage, Germany; #1320).

### Human tissue

Full-thickness human duodenum biopsies used for small intestinal crypt isolation were obtained from obese patients undergoing a stomach bypass operation (*n* = 4; Supplemental Table 1) at the surgery unit of the University Hospital Würzburg. The study was approved by the Institutional Ethics Committee on human research of the Julius-Maximilians University Würzburg (approval number 182/10). Informed written consent was obtained before surgery and data analysis was done anonymously according to the principles expressed in the “Declaration of Helsinki.”

### Isolation and expansion culture of small intestinal spheroids/organoids from primary tissue

Intestinal crypts were isolated from the murine intestine or human biopsies (Supplemental Table 1) and cultured as previously described.^8,14,18^ Briefly, the crypt containing epithelial tissue was incubated after explantation without the villus domain in 2 mM EDTA/HBSS− solution (Sigma-Aldrich, St. Louis, MO) for 30 min at 4°C under gentle rotation. Subsequently, the tissue was transferred into 10 mL fresh HBSS− solution and manually shaken for 2 min. This step was repeated three times, each time in fresh HBSS− solution with increasing force. Afterwards, the crypt-containing fractions were pooled, centrifuged at 350×*g* for 3 min and the pellet was resuspended in 450–900 µL of cold Matrigel® (Corning, Hickory, NC) depending on pellet size. Droplets of 50 µL were plated per well in a 24-well plate and incubated at 37°C until solidification of Matrigel®. Subsequently, Matrigel® drops were covered with 500 µL spheroid maintenance medium (SM medium; Supplemental Tables 3 and 4). After growth of crypts into spheroidal cell clusters comprising mainly proliferative stem and progenitor cells of the intestinal epithelium, cultures were collected in a falcon tube, placed for 30 min on ice and centrifuged at 350×*g* for 3 min. Next, pellet was dissociated by incubation in 1–2 mL 1× TrypLE express (Thermo Fisher Scientific, Waltham, MA) for up to 10 min at 37°C. To stop the enzymatic digestion, 2–3 mL crypt medium (Supplemental Tables 3 and 4) were added. For cell expansion, dissociated spheroids were washed in crypt medium and embedded in Matrigel® droplets (50 µL per well of a 24 well plate) that were covered with 500 µL SM medium (Supplemental Tables 3 and 4) after solidification. Cells were cultured at 37°C, 95% humidity, 5% CO_2_. Splitting procedure was repeated once or twice a week, depending on cell density. For intestinal organoid cultures exhibiting distinct differentiated intestinal cell entities of the secretory and absorptive lineage,^19^ spheroids were cultured for at least 7 days in SM medium (Supplemental Tables 3 and 4).

### Immortalization and expansion culture of immortalized cell lines

Immortalization was performed by InSCREENeX GmbH Braunschweig on the murine/human intestinal organoid cultures as previously described.^20–22^ In brief, HEK293T cells were transfected with a cocktail consisting of three packaging plasmids encoding the helper functions (gagpol, rev, and VSVg) and one of the twelve lentiviral vectors which are listed in Supplemental Table 2 to produce the lentiviral supernatant for immortalization. After 48 h, lentivirus containing supernatant was collected and concentrated before organoids were transduced by one of two strategies: (1) transduction in 2D: primary intestinal epithelial cells derived from the established organoid lines were cultured as single cells on fibronectin/collagen-I coated tissue culture plates or (2) transduction in 3D: organoids were isolated from Matrigel® droplet cultures and transferred into suspension culture for transduction in floating conditions.

The viral cocktail used for immortalization contained in total 10 genes for transduction of murine intestinal organoids (Supplemental Table 2; genes with no star) and 12 genes for transduction of human intestinal organoids (Supplemental Table 2). To increase transduction efficiency, 0.25% polybrene was added to the cell culture medium for 24 h. The next day, medium was changed for fresh murine/human SM medium (Supplemental Tables 3 and 4) without polybrene. The 2D transfected cells were cultured for several days until formed colonies were picked and expanded on fibronectin/collagen-I coated tissue culture plates. The 3D transfected cells were transferred to Matrigel® drop cultures 24 h after polybrene treatment and were cultured until colonies grew out of the Matrigel® drop followed by hand picking of cells and 2D expansion culture on fibronectin/collagen-I coated plates.

Cell cultures were pre-screened on behalf of their epithelial-like morphology and/or their proliferative capacity. By this, three murine and one human cell line were established that were cultured in cell line-specific medium (m/h CL medium; Supplemental Tables 3 and 5). While the murine cell lines (I9K6, I9K8, and I12K9) were cultured on untreated tissue culture plastic with a split ratio of 1:10–1:20, the human cell line (15-06 I4B) was expanded on collagen-I coated tissue culture flasks (coating with collagen-I (rat-tail) acetic acid solution 0.4% for 30 min; isolation in-house from rat tails) using split ratios of 1:2–1:3. In general, cell lines were split by incubation with 1× Trypsin/EDTA in PBS− (without Mg^2+^ and Ca^2+^; Sigma-Aldrich, St. Louis, MO) for 5 min at 37°C and enzymatic reaction was stopped with 10% FCS. Characterization analyses of the established cell lines were performed from passage 10–20 on.

### Intestinal Tissue Engineering in Transwell®-like cell culture formats

For establishing scaffold-based in vitro models of the small intestine on Transwell® systems, commercially available 24 well plate polyethylene terephthalate (PET) inserts (pore size 1.0 µm; 662610, Greiner Bio-One GmbH, Frickenhausen, Germany) were coated with 0.1 mg/mL collagen-I solution (isolation in-house from rat tails) as previously described.^23^ Briefly, inserts were seeded with 5 × 10^4^ single cells, followed by medium change every 2–3 days with 300 µL CL-specific medium (Supplemental Tables 3 and 5) in the apical and 900 µL in the basolateral compartment. Experimental assays were performed on day 14 for the human 15-06 I4B line and on day 21 for the murine cell lines (I9K6, I9K8, and I12K9), as the measured barrier integrity (TEER) values remained stable for later time points (data not shown).

### Flow cytometry

Proliferation rates of the established cell lines were determined by using the EdU Click-it® kit (Life Technologies, Darmstadt, Germany) according to the manufacturer’s instructions. Cell lines cultured as monolayers in 2D were analyzed when achieving 50% confluence, while cell lines cultured in 3D Matrigel® droplets were analyzed 3 days after splitting. Briefly, cells were incubated for 2 h at 37°C in pre-warmed CL medium (Supplemental Tables 3 and 5) supplemented with 1 mM EdU before harvesting as single cells and washing in PBS− containing 1% Bovine Serum Albumin (BSA; Sigma-Aldrich, St. Louis, MO). Afterwards, cells were fixed for 15 min at room temperature (RT) in the dark with 100 µL Click-it® fixative, washed in 1% BSA/PBS− solution and permeabilized in 100 µL of a 1× saponin-based buffer (15 min, RT, protected from light). After incubation for 30 min at RT with 500 µL of the EdU Click-it® Plus reaction cocktail containing Alexa Fluor® 647 as fluorescent dye, the samples were washed in 1× saponin-based buffer and analyzed on the BD FACS Accuri.

### Histology and immunofluorescence

For histological and immunofluorescence analyses, cells cultured as monolayers on tissue culture plates or on Transwell®-inserts were fixed with 4% paraformaldehyde (Roti-Histofix 4%, Carl Roth, Karlsruhe, Germany) for 1 h at 4°C, while organoid cultures set up in 3D were first isolated from Matrigel® droplets. For this, droplets were resuspended in the surrounding medium and the organoid suspension was collected into a falcon tube. Organoids were centrifuged at 350×*g* for 3 min with subsequent washing in PBS− before fixation for 30 min at 4°C occurred. After fixation, samples were washed in PBS− before Transwell®-inserts and 3D-cultures were covered with Histogel (VWR, Wayne, PA, USA), followed by paraffin embedding. For (immuno-)histological analyses of Transwell®-inserts and 3D-cultured organoids, tissue sections of 5 µm thickness were prepared. Hematoxylin and eosin staining (H&E; Morphisto, Frankfurt am Main, Germany) was performed according to standard protocols.^24^ Immunohistochemical analyses required deparaffinization of the tissue sections followed by rehydration before antigen retrieval by incubation at 100°C for 20 min in citrate buffer (pH 6; Roth, Karlsruhe, Germany). In contrast, 2D monolayer cultures were treated with 0.2% Triton-X100 (Sigma-Aldrich, St. Louis, MO, USA) in PBS− for 30 min at RT and washed in PBS− + 0.5% Tween-20 for 5 min on a shaker. After pre-treatment, all samples were blocked for 30 min with 5% donkey serum in PBS− and incubated with primary antibodies (Supplemental Table 6) overnight at 4°C. On the next day, samples were washed three times for 5 min each, followed by incubation with secondary antibodies (Supplemental Table 6) for 1 h in the dark at RT. Subsequently, samples were washed three times for 5 min each, fixed on glass slides, and covered with Fluoromount-G with 4′,6-diamidino-2-phenylindole (DAPI; Thermo Fisher Scientific, Waltham, MA) to stain cell nuclei. Confocal (Leica TCS SP8, Leica, Wetzlar, Germany) or epifluorescence inverted microscopy (Keyence BZ-9000, Japan) was used for imaging.

For ultrastructural analyses, Transwell® models were fixed either in 2.5% glutaraldehyde for transmission (TEM) or 6.5% glutaraldehyde for scanning electron microscopy (SEM) imaging. Sample processing and imaging was performed at the core facility of Prof. Dr. Stigloher (Julius-Maximilians-University Würzburg). Images were processed using the ImageJ software (National Institutes of Health, Bethesda, MD).

### Barrier integrity measurement

The formation of a tight epithelial barrier was investigated by transepithelial electrical resistance (TEER) measurements and Fluorescein isothiocyanate (FITC)-dextran (Sigma-Aldrich, St. Louis, MO) permeability studies. Every 2–3 days over a culture time of 21 days for murine and 14 days for human cell-line based Transwell®-models, TEER-values were measured by using a commercially available hand-electrode (Millicell ERS-2; Millipore, Billerica, MA). As endpoint analyses for the respective models, a FITC-dextran (10 µM; 4 kDa) assay was performed as previously described.^14,23^ The FITC-dextran permeability rate was determined in percent.

### Transport studies

Epithelial transport studies were performed according to Fey et al.^23^ using the reference substances fluorescein sodium salt (10 µM; paracellular transport), propranolol hydrochloride (100 µM; transcellular transport), and rhodamine123 (100 µM; efflux-transporter *p*-glycoprotein Mdr1; all Sigma-Aldrich, St. Louis, MO), each dissolved in cell line-specific culture medium (Supplemental Table 3). Briefly, reference substances (300 µL) were applied apically and samples of 100 µL were taken every 15 min for 2 h from the basolateral compartment (900 µL), followed by replacement with 100 µL fresh medium. Fluorescence intensity of fluorescein sodium salt and rhodamine123 was determined with a microplate reader (Tecan Infinite M200, Maennedorf, Switzerland) and propranolol hydrochloride was analyzed by HPLC-MS/MS (Shimadzu Nexera LC-30AD, CTO-20AC with FCV-12AH, 8030 Plus; Shimadzu Corporation, Kyoto, Japan). HPLC-MS/MS analysis was performed by the Sapiotec GmbH in Würzburg. The apparent permeability coefficient (P_app_) was calculated according to Artursson et al.^9,25^

### Quantitative real-time PCR

For RT-qPCR, cellular monolayers grown on tissue culture plastic or on Transwell®-inserts were treated with RLT buffer complemented with 1% 2-mercaptoethanol, while 3D organoid cultures were isolated from Matrigel® drops as before. Total RNA was extracted after homogenization through a QIAshredder spin column with the RNeasy micro Kit (Qiagen) including the DNase treatment for 15 min at RT. Next, cDNA was produced from 1 µg RNA using the i-Script Reverse transcription Supermix (Bio-Rad), followed by real-time quantitative PCR (RT-qPCR) with 1 µL cDNA using the SsoFast^TM^ EvaGreen® Supermix and a CFX 96 Touch^TM^ RT-qPCR Detection System (Bio-Rad). Primer pairs are listed in Supplemental Table 7. As reference genes, *mRpl15* and *mRps29* were used for murine cells and *hHPRT1* and *hEF1α* for human cells. Data were analyzed according to D’Antonio et al.^26^

### MicroArray analysis

For MicroArray analysis, 5 × 10^5^ cells/mL were seeded and incubated until the murine cell lines were grown confluently (2 days). For the human cell line Transwell®-models were used after 14 days of culture. Total RNA was isolated using the miRNeasy® kit (QIAGEN) according to the manufacturer’s instructions. Subsequent to RNA purification, the TURBO DNA-free^TM^ kit was used to remove genomic DNA from RNA samples. Samples were controlled for purity and integrity on the Agilent Technologies 2100 Bioanalyzer (Agilent Technologies). Only RNA with RIN (RNA integrity number) values greater than 8 were used for further analysis.

About 2–10 ng of total RNA were used for biotin labelling according to the GeneChip® Pico kit protocol (Affymetrix). Biotinylated cDNA were fragmented and placed into a hybridization cocktail containing four biotinylated hybridization controls (BioB, BioC, BioD, and Cre) as recommended by the manufacturer. Samples were hybridized to an identical lot of Affymetrix Clarion^TM^ S (400 format) for 17 h at 45°C. Hybridization was performed for 16 h at conditions recommended by the manufacturer. Clariom^TM^ S chips were washed and stained in the Affymetrix Fluidics Station 450.

Gene Chips were scanned using the Affymetrix GCS 3000. Image analysis was done by Affymetrix® GeneChip® Command Console® Software (AGCC) and Affymetrix® Expression Console^TM^ Software.

### MicroArray data analysis

Raw data obtained after image analysis were analyzed by R/BioConducter packages “oligo” and “Biobase.” Raw signal intensities of each probe set (gene feature) were summarized by median polish method. Summarized probe set data was log2 transformed followed by RMA normalization procedure. Finally, the obtained data set was annotated by NetAffx (Affymetrix). Normalized data sets were filtered for informative genes (showing at least expression values >log2(10) in more than two samples). Datasets were tested across all groups (ANOVA) or pairwise using linear models to assess differential expression in context of the multifactorial designed experiment. For statistical analysis and assessing differential expression, the R/BioConductor package “limma” was used which utilizes an empirical Bayes method to moderate the standard errors of the estimated log-fold changes.^27^ Volcano plots of differentially expressed genes and clustergrams were generated using MATLAB (MathWorks Inc., Las Vegas, NV) Bioinformatics Toolbox. Normalized gene expression data were analyzed using the clustergram function to identify differential clustering of the cell lines based on intestinal epithelial cell markers, using the Euclidean distance as a clustering parameter. Gene enrichment analysis and pathway enrichment network was conducted in Cytoscape (Institute of Systems Biology, Seattle, WA).^28^ For gene enrichment analysis the plug-in ClueGO^29^ was used to identify the most significantly enriched biological processes. Pathway enrichment network was generated by applying a query of the significantly differential expressed genes in Reactome and networks were generated in Cytoscape software (Institute of Systems Biology, Seattle, WA).

### Statistical analyses

Statistical significance was determined using one-way ANOVA, Tukey’s multiple comparison test by GraphPad Prism 6 Software (GraphPad Software, Inc., La Jolla, CA). Results are displayed as mean ± standard deviation (SD). The level of statistical significance is indicated as follows: *p < 0.05, **p < 0.01, ***p < 0.001.

## Results

### Lentiviral transduction of organoid cultures with a defined set of genes results in the establishment of permanent cell lines

**Figure 1.**
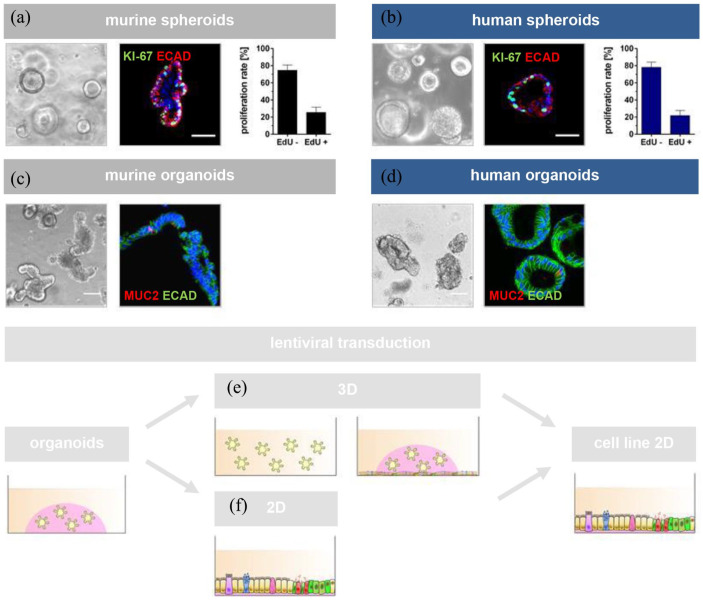
Characterization of intestinal spheroid cultures and experimental strategy of immortalization. Murine and human intestinal spheroid cultures (a+b) characterized for cell type-specific key features prior to induction of differentiation. IHC analyses demonstrated the presence of Ki-67 positive cells (green) representative of proliferative stem and/or transit amplifying precursor cells. Red IHC signals revealed the expression of ECAD, an adherence junction marker of intestinal epithelial cells in general. DAPI counterstaining (blue) marked cell nuclei. Quantitative analyses of proliferation rates measured by flow cytometry demonstrated rates of around 20% EdU positive cells which is characteristic for intestinal spheroid cultures. Murine and human intestinal organoid cultures (c+d) were further characterized for cell type-specific key features prior to immortalization. IHC analyses showed the presence of MUC2 positive cells (red) representative for mucus-producing goblet cells. ECAD (green) and DAPI (blue) staining demonstrated adherence junctions and cell nuclei, respectively. *n* = 3. Scale bar = 50 µm. Graphical abstract (e+f) of the lentiviral transduction strategy performed under either 3D floating conditions (e: cells cultured as organoids in suspension culture followed by Matrigel embedding) or on 2D cultures (f: single cells grown as monolayer cultures). Established cell lines (three murine/one human) were cultured as monolayers on fibronectin/collagen-I coated plates in 2D. IHC: immunohistochemistry; ECAD: E-Cadherin; EdU: 5-ethynyl-2′-deoxyuridine; DAPI: 4′,6-diamidino-2-phenylindole; MUC2: Mucin-2.

Intestinal spheroid cultures from murine and human intestinal crypts (Supplemental Table 1) were established according to protocols published by Sato et al.^8,18^ Due to prolonged culture time, spheroid lines from four different murine and four distinct human donors were differentiated into organoids, thereby representing differentiated intestinal cells of the secretory and absorptive lineage.^19^ Key features of primary spheroid cultures (Figure 1(a+b)) were analyzed before differentiation toward organoids used for immortalization, demonstrating a characteristic proliferative potential depicting Ki-67 positive cells (Figure 1(a+b)) with a mean proliferation rate of 25.5 ± 6.2% for murine (Figure 1(a)) and 21.6 ± 6.0% for human spheroids (Figure 1(b)). In addition, primary organoid cultures (Figure 1(c+d)) were histologically analyzed before immortalization, indicating differentiated intestinal cells of the secretory lineage, as MUC2 positive cells were identified in immunohistochemistry (IHC) staining of Histogel®-embedded murine and human organoid cultures (Figure 1(c +d)).

The gene transduction strategy was performed either under 3D-floating conditions (Figure 1(e) or after culture on fibronectin/collagen-I pre-coated plates in 2D (Figure 1(f)) to increase the immortalization efficiency. Cell cultures were transduced with lentiviruses encoding a 10-component gene pool for murine cells and a 12-component gene pool for human cells (Supplemental Table 2). The respective genes integrated by lentiviral transduction are highlighted (bold = murine and human; bold + star = human only) for the immortalized primary organoid-based cell lines in Supplemental Table 2. Colonies were picked manually after transduction to establish clonal cell lines. Based on the previously defined criteria such as culture conditions, cellular morphology, or proliferation capacity, three murine cell lines (termed I9K6, I9K8, and I12K9) and one human cell line (termed 15-06 I4B) were successfully generated as alternative intestinal epithelial cell lines to commonly known cell lines such as Caco-2.

Subsequently, we re-introduced the culture conditions for the newly established cell lines compared to the culture conditions known for primary organoid cultures. Intestinal organoids are routinely setup in a 3D environment embedded in Matrigel® together with the supplementation of growth factors mimicking niche conditions for instance to support ISC and transit amplifying cell (TAC) proliferation (R-Spondin1), to inhibit differentiation (Noggin), to regulate cell migration (EGF) and to prevent apoptosis (Y-27632) in vitro.^2,18,30^ The immortalized murine cell lines I9K6, I9K8, and I12K9 enabled a more time- and cost-efficient culture, as these cells grew on uncoated standard plastic material (Supplemental Figure 1) and were dependent on growth factor supplementation of the basic cell culture medium only supplemented with 10% FCS, hEGF, mNoggin and Y-27632 (Supplemental Figure 2). R-Spondin1 as most cost-intensive growth factor could be excluded without losing growth characteristics as demonstrated in morphological and quantitative cell growth analyses (Supplemental Figure 2). For the established human cell line, we showed similar advantages in the context of cost-efficient culture conditions, as these cells grew on collagen-I pre-coated surfaces (Supplemental Figure 1) and therefore complex splitting procedures and associated high costs of 3D cultures were avoided. However, in the context of simplified and cost-efficient medium formulations no adaptions could be applied for the established human line 15-06 I4B (Supplemental Figure 2).

### Primary organoid-based cell lines demonstrate infinite cell growth

**Figure 2.**
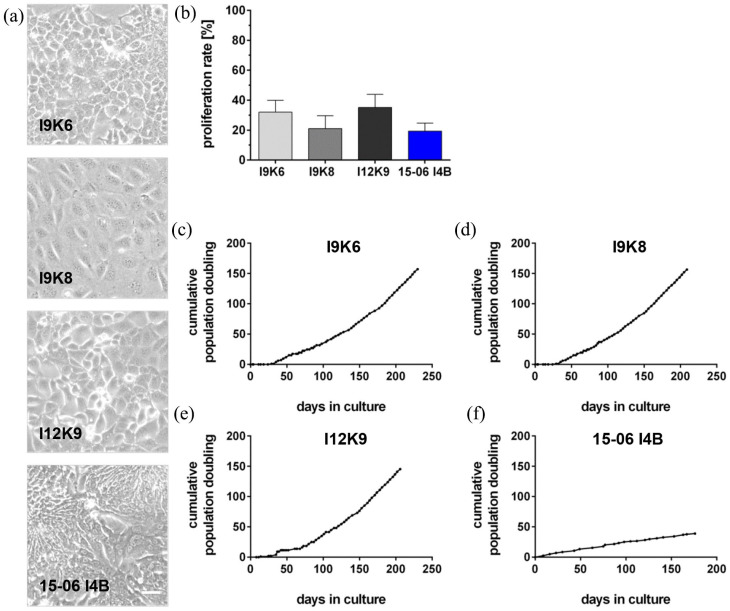
Murine and human cell lines show indefinite proliferation demonstrating cell line characteristics. Representative microscopic images of murine (I9K6, I9K8, and I12K9) and human cell lines showed an epithelial-like cell morphology over 8–10 passages in culture (a), even though 15-06 I4B cells revealed a more elongated cell morphology. Scale bar = 100 µM. Proliferation capacity was monitored in a quantitative EdU proliferation assay using flow cytometry (b). The respective assay was performed every five passages starting with passage 10. Cumulative population doubling analyses (>200 days) showed an exponential cell growth for I9K6 (c), I9K8 (d), and I12K9 (e) cells, while the human cell line 15-06 I4B (f) demonstrated a linear cell growth. *n* = 3. EdU: 5-ethynyl-2′-deoxyuridine.

The newly generated murine cell lines called I9K6, I9K8, and I12K9 as well as the human cell line termed 15-06 I4B were characterized in long-term culture as cell lines with an infinite cell growth. Their respective morphological characteristics with an epithelial-like cell appearance (Figure 2(a)) did not change during the applied culture time (8–10 passages). Proliferation rate analyses by EdU flow cytometry (Figure 2 (b)), starting from passage 10 of the 2D cultured cells, with three independent replicates every five passages, demonstrated an ongoing high proliferation capacity in long-term culture (Table 1). The mean proliferation rate of I9K8 and 15-06 I4B cells was similar to primary organoids, while I9K6 and I12K9 showed higher proliferation rates. In addition, these cell lines demonstrated indefinite proliferation without any signs of senescence for up to 200 days (Figure 2(c–f)); however, exponential cell growth was only observed for the murine cell lines (Figure 2(c-e)).

**Table 1.** Proliferation rate determined by EdU-Click it® assay.

	Proliferation rate [%] 2D-culture		Proliferation rate [%] 3D-culture	
	EdU−	EdU+	EdU−	EdU+
I9K6	68.0 ± 8.0	32.0 ± 8.0	91.2 ± 3.1	8.8 ± 3.1
I9K8	78.9 ± 8.5	21.1 ± 8.5	88.4 ± 5.9	11.6 ± 5.9
I12K9	64.8 ± 8.7	35.2 ± 8.7	88.3 ± 0.7	11.7 ± 0.7
15-06 I4B	80.6 ± 5.3	19.4 ± 5.3	84.7 ± 3.2	15.3 ± 3.2

Rate given in percent for murine and human cell lines cultured in 2D and 3D.

### Immortalized cell phenotype predominantly consists of differentiated epithelial cell types

**Figure 3.**
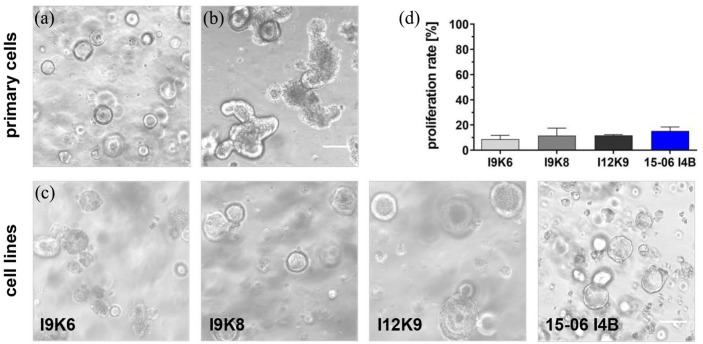
Cell lines form proliferative spheroid-like 3D structures in a 3D-Matrigel® environment. Murine and human primary epithelial cells formed spheroids (a) and organoids (b), depending on the culture conditions. Representative microscope images demonstrated the formation of spheroid-like structures also for the dissociated cell lines I9K6, I9K8, I12K9, and 15-06 I4B when embedded into Matrigel® droplets (c). Scale bar = 100 µm. Proliferation rate was measured by an EdU assay using flow cytometry (d). *n* = 3. EdU: 5-ethynyl-2′-deoxyuridine.

Crypts composed of mainly intestinal stem cells (ISCs) form spheroids under proliferative culture conditions when embedded in a 3D-environment (Figure 3(a)). Due to prolonged culture time, spheroids transformed into differentiated organoids (Figure 3(b)), thereby representing the cellular composition as well as the structural organization of the intestinal epithelium. Given that differentiated organoid cultures were used for lentiviral transduction, the generated cell lines were investigated for their capacity to re-aggregate into 3D structures and to demonstrate a similar structural organization to primary organoids. As shown in Figure 3(c), representative microscopic images demonstrate the re-aggregation into 3D structures with a spheroid-like morphology for all cell lines when cultured under similar conditions known for primary organoids. An extended culture time did not contribute to the formation of an organoid-like cytoarchitecture with characteristic separation into crypt-villus-domains. However, cell aggregates were proliferative (Figure 3(d)). The proliferation rate was measured on day 3 after splitting of spheroids to single cells followed by re-embedding in Matrigel® drops. As shown in Table 1, only minor differences were observed between the established murine and human spheroid-like cell clusters, but a decreased proliferation capacity was identified compared to 2D cultures. Taken together, a reduced proliferation rate and the lack of capability to form an organoid-like cytoarchitecture supports the generation of an immortalized phenotype for all cell lines.

**Figure 4.**
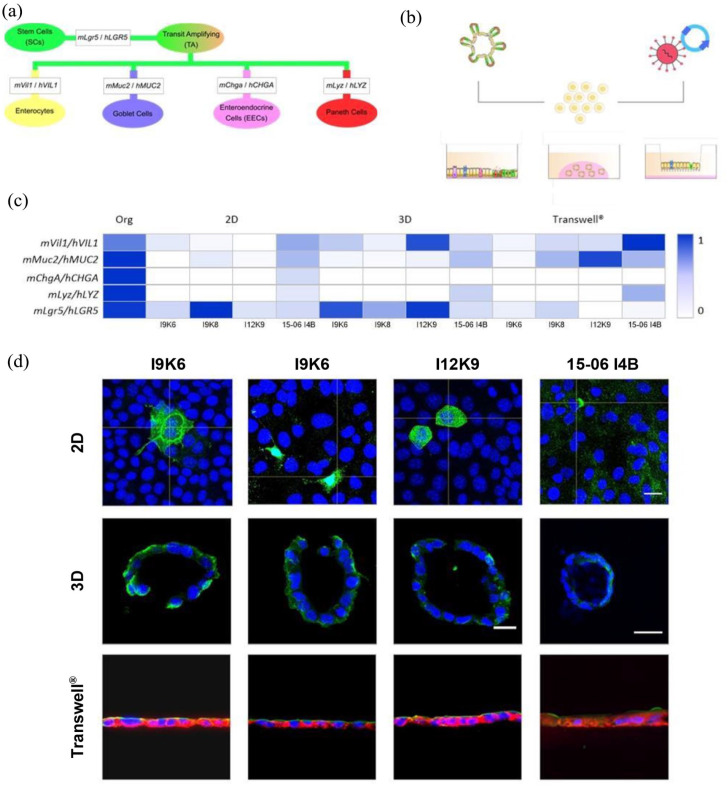
Cell lines exhibit marker genes specific for individual cell-types localized in the intestinal epithelium indicating the preservation of native features after immortalization. Markers representative for the cell types found in the intestinal epithelium were analyzed (a). The generated murine and human cell lines were cultured in 2D, 3D (Matrigel®) and on Transwell®-inserts after immortalization by lentiviral transduction of organoids (b). Gene expression profile was investigated by RT-qPCR and set in relation to the reference genes *mRpl15* and *mRps29* for murine cells as well as *hHPRT1* and *hEF1*-α for human cells. The mRNA transcript levels obtained for murine and human organoids were set to 1 (c). Immunohistochemistry analyses of 2D and 3D cultured cell lines as well as cultures setup on Transwell®-inserts (d). Representative microscopic images show the protein expression pattern of VIL1 (green). ECAD (red) and DAPI (blue) were stained to identify the cell borders and the cell nuclei. *n* = 3; Scale bar = 20 µm (2D; 3D, Transwell®); Scale bar = 50 µm (3D, 15-06 I4B). qRT-PCR: quantitative Real-Time Polymerase Chain Reaction; HPRT1: hypoxanthine phosphoribosyltransferase; EF1-α: elongation factor-1 alpha; Rpl15: ribosomal protein L15; Rps29: ribosomal protein S29; ECAD: E-Cadherin; DAPI: 4′-6-diamidio-2-phylindole; RNA: ribonucleic acid; VIL1: Villin-1.

To further validate the differentiated phenotype of the established cell lines, specific gene and protein expression profiles of intestinal epithelial cells, were investigated. The marker panel comprised Villin-1 (Vil1) for enterocytes, Mucin-2 (Muc2) for goblet cells, Chromogranin-A (Chga) for enteroendocrine cells, Lysozyme (Lyz) for Paneth cells and Leucine rich repeat containing G protein-coupled receptor 5 (Lgr5) for remaining stem cell signatures (Figure 4(a)). In addition, expression profile influencing factors, for instance 2D or 3D culture conditions as well as Transwell®-like cultures with semipermeable membranes were taken into account and analyzed by RT-qPCR and IHC (Figure 4(b)).

All four lines (of murine and human origin) individually expressed the specific marker genes, albeit at reduced levels when compared to primary organoid cultures (Figure 4(c)). However, *mChga/hCHGA* was not detected for the different cell lines or different culture conditions. As shown in Figure 4(c), the mRNA transcript level of *mLgr5* for murine cell lines was reduced in Transwell®-based systems, while 3D cultures demonstrated an increase compared to 2D cultures. For *mMuc2* and *mVil1*, we observed an increase in the gene expression level when cultured on Transwell®-inserts, at least for I9K8 and I12K9 cells and also an increase of *mVil1* in 3D cultures in relation to the 2D cultures of all murine cell lines. A similar observation was made for the human cell lines, as the mRNA transcript level of *hVIL1*, *hMUC2*, and *hLYZ* was increased in Transwell®-based cultures compared to 2D cultures. In addition, our established models showed a reduced level of *hLGR5* when cultures were set up on semipermeable membranes. Interestingly, the mRNA transcript level of *hLYZ* increased together with culture complexity, as shown in Figure 4(c) for 3D and Transwell®-based cultures (Figure 4(c)).

Next, the corresponding protein expression pattern was studied for the 2D, 3D as well as Transwell®-based cultures (Figure 4(d)). IHC analyses revealed signals for Villin-1 with individual signal intensities. While IHC signals for Villin-1 were weak in 2D cultures, cross-sections of Transwell®-based cultures demonstrated for all cell lines a signal of Villin-1 on the apical cell surface, giving a hint for a polarized epithelial monolayer. Interestingly, spheroids of the respective clones revealed a partially outward-facing staining of Villin-1, instead of the known internal protein expression pattern in primary organoids, thereby indicating a loss of inward polarization in spheroid-like 3D structures (Figure 4(d)).

### Primary-organoid-based cell lines form a tight and characteristic epithelial cell layer

**Figure 5.**
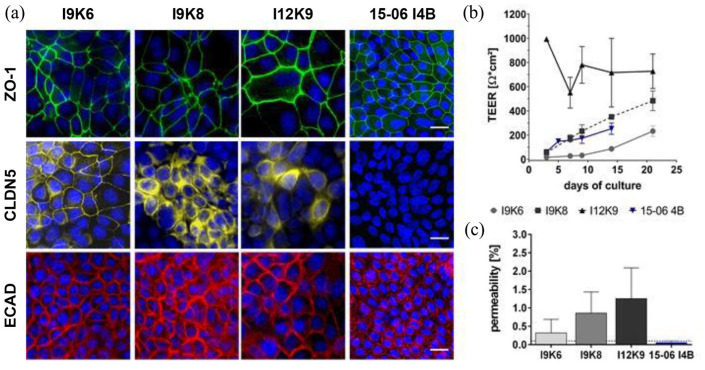
Histological and functional characterization of epithelial barrier function. Primary-organoid-based cell lines of murine (I9K6, I9K8, and I12K9) and human origin (15-06 I4B) were seeded on either uncoated plastic (murine cell line) or Col I pre-coated plastic (human cell line) until a confluent layer was formed. Tight/adherens junction proteins ZO-1 (green), CLDN5 (yellow), and ECAD (red) showed an equal expression by IF-staining in representative microscopic images (A). The human cell line demonstrated no expression for CLDN5 by IF-staining (a). Cell nuclei are counterstained with DAPI (blue) (a). Scale bar = 20 µM. PET-based Transwell® models were used for TEER and permeability studies. TEER was monitored for up to 14 days for 15-06 I4B and for up to 21 days for all three murine cell lines (b). Permeability studies were performed by FITC-dextran measurements for I9K6, I9K8, I12K9, and 15-06 I4B cell-based models (c). *n* = 3. TEER: Transepithelial electrical resistance; PET: polyethylene terephthalate; FITC: fluorescein isothiocyanate; ECAD: E-Cadherin; CLDN5: Claudin-5; ZO-1: zonula occludens-1; DAPI: 4′,6-diamidino-2-phenylindole; Col I: collagen-I.

Intestinal epithelial in vitro models are characterized by the formation of a tight barrier, comprising tight and adherens junction proteins such as Zonula occludens-1 (ZO-1), Claudin-5 (CLDN5), or E-Cadherin (ECAD).^23,31^ Therefore, IF staining’s were performed for the murine cell lines (I9K6, I9K8, and I12K9) after a culture time of 5 days on uncoated plastic and for the human cell line (15-06 I4B) after 7 days on Col I pre-coated plates. A characteristic tight/adherens junction protein expression pattern was shown by ZO-1 and ECAD for all cell lines when a confluent cell layer was formed (Figure 4(a)). In contrast, CLDN5 demonstrated a continuous expression pattern only for I9K6 cultures, while I9K8 and I12K9 cells revealed a more scattered and inhomogeneous IHC signal (Figure 5(a)). With regard to the human cell line 15-06 I4B, we could not observe an expression pattern for CLDN5 in our studies, indicating that CLDN5 expression was abrogated after immortalization in these cells (Figure 5(a)).

Next, we proved the capacity of our established murine and human cell lines to generate an intact and functional barrier. Such characteristics are prerequisites to demonstrate an alternative intestinal in vitro model to commonly used Caco-2 cell-based models for pre-clinical applications. Therefore, cell lines with a seeding density of 5 × 10^4^ cells per/24 Transwell® insert were cultured on Col I pre-coated PET membranes and transepithelial electrical resistance (TEER) values were measured with a non-destructive TEER electrode every 2–3 days (Figure 5(b)). A tight barrier was determined after a culture time of 21 days for murine and 14 days for human cell lines (Figure 5(b)). While the human cell line 15-06 I4B (mean maximum 253 ± 47 Ω cm²) and the murine cell line I9K6 (mean maximum 232 ± 44 Ω cm²) showed a rather similar and relatively low TEER value, the murine cell line I9K8 with a mean maximum of 483 ± 81 Ω cm² and the cell line I12K9 with a mean maximum of 727 ± 143 Ω cm² demonstrated comparatively higher TEER values. In addition, the formation of a tight barrier was shown by permeability rates of less than 2% for 4 kDa FITC-dextran molecules in models set up with 15-06 I4B (0.06 ± 0.05%), I9K6 (0.32 ± 0.37%), I9K8 (0.86 ± 0.57%) and I12K9 (1.26 ± 0.83%) cells, as shown in Figure 5(c).

### Primary organoid-based cell lines show organ-specific transport functions

**Figure 6.**
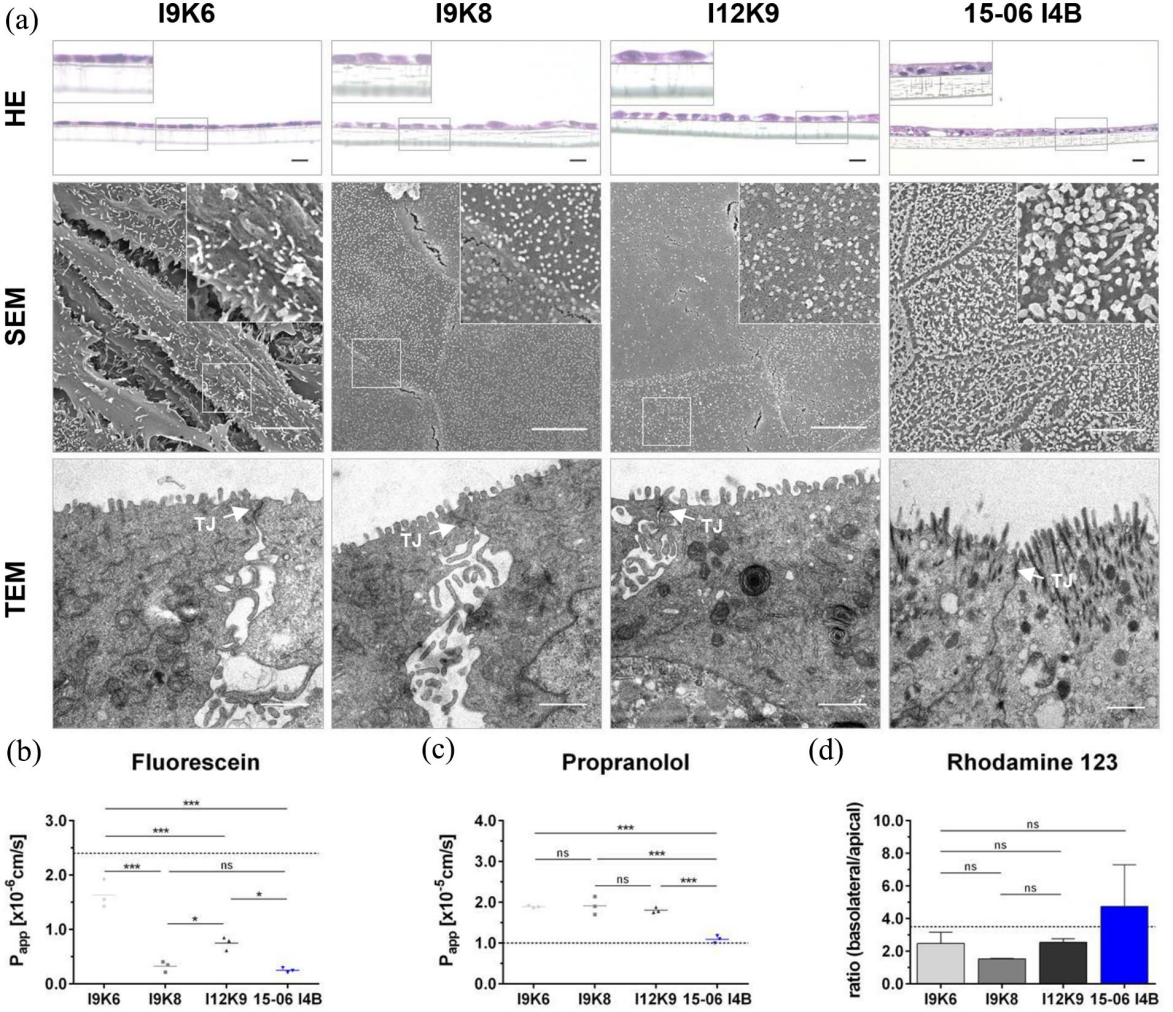
Cell lines cultured on Transwell® inserts formed a functional monolayer with tight junctions, microvilli, and a characteristic transport capacity. Cross-sectional images of H&E-stained models show a closed cell layer after a culture time of 21 days for murine cell lines (I9K6, I9K8, and I12K9) and after 14 days for the human cell line 15-06 I4B (a). The formation of a consistent cell layer with apical microvilli and tight junctions (TJ; arrow) was demonstrated in representative SEM and TEM images (a). Scale bar in HE murine = 20 µm; HE human = 200 µm; in SEM = 5 µm; in TEM = 1 µm; representative of *n* = 3 biological replicates. Paracellular transport of fluorescein was lower in I9K8 and 15-06 I4B cell line-based models (b). Propranolol transport (transcellular transport) was higher in murine cell line-based models compared to the human cell line-based models (c). Rhodamine 123 efflux-transport capacity for the murine cell line-based models was lower compared to the human cell line-based model (d). Transport capacities of Caco-2 Transwell®-models are presented as reference values (dotted line; b–d).^23^ Significance was calculated by one-way ANOVA. P_app_: apparent permeability coefficient; SEM: scanning electron microscopy; TEM: transmission electron microscopy; H&E: hematoxylin and eosin; TJ: tight junctions. *n* = 3. *p < 0.05. **p < 0.01. ***p < 0.001.

Primary organoid-based cell lines formed a tight epithelial monolayer together with a characteristic tight/adherens junction protein expression profile when setup on a Col-I coated surfaces. An integer barrier, as one of the main features of the intestinal epithelium, was investigated by histological and ultrastructural analyses as well as transport capacity studies. Representative microscopic images of H&E-stained sections (Figure 6(a); HE) of Transwell®-based murine cell cultures (I9K6, I9K8, and I12K9) revealed a dense as well as flat, elongated cellular monolayer. In addition, a weak eosin staining was shown, thereby indicating a less compact connective tissue. In contrast, the human cell line 15-06 I4B showed a densely packed, flat cell cluster surrounded by compact connective tissue in H&E-stained sections (Figure 6(a); HE). High-resolution SEM and TEM analyses likewise showed the formation of a consistent cell layer (Figure 6(a); SEM, TEM). Characteristic tight junctions (TJs; marked with an arrow) and a brush border membrane with apical microvilli were also demonstrated by ultrastructural analyses. Transwell® cultures based on the human cell line showed an even tighter distribution as well as longer microvilli on the apical cell surface compared to murine cell line cultures I9K6, I9K8, and I12K9 (Figure 6(a); TEM), thereby demonstrating a better similarity to the in vivo structure of the small intestinal epithelium.

To investigate the transport activity of the established Transwell® cultures, reference substances characterizing the paracellular-, transcellular-, and efflux-transport routes were applied. Fluorescein, serving as a reference substance (low-permeable) for paracellular transport, showed a six-times lower permeability for 15-06 I4B (*P*_app_-value of 2.5 × 10^−7^ cm/s) or I9K8 (*P*_app_-value of 3.2 × 10^−7^ cm/s) cell line-based Transwell®-models compared to models set up with I9K6 cells (*P*_app_-value of 1.6 × 10^−6^ cm/s), and a two-times lower permeability compared to I12K9 (*P*_app_-value of 7.5 × 10^−7^ cm/s) cell line-based models, indicating the tightest barrier for models based on 15-06 I4B and I9K8 cells (Figure 6(b)). Models based on I9K6, I9K8, and I12K9 cells showed a similar permeability for propranolol in transcellular transport studies, while the human cell line-based models (*P*_app_-value of 1.1 × 10^−5^ cm/s) demonstrated a significantly reduced transport capacity for propranolol. The reference substance rhodamine 123 was applied either to the apical or the basolateral compartment of the established models, as the efflux transport mechanism is described as the basolateral/apical ratio (ba/ab = basolateral-apical/apical-basolateral). A similar ratio was observed for models based on I9K6 or I12K9 cells (ba/ab = 2.5), while a slight decrease was demonstrated for I9K8 cell-based models (ba/ab = 1.5) as shown in Figure 6(d). Of note, human cell line-based models showed the highest efflux ratio (ba/ab = 5.1; Figure 6(d)). Results observed for Caco-2 cell-based models are represented by a dotted line in the respective graphs (Figure 6(b)—(d)).^23^ Taken together, these data indicated the formation of a tight and functional epithelial barrier with partly improved transport functions compared to the as “gold standard” known Caco-2-based cultures.

### Similarity assessment of the gene expression profile of two different murine cell lines

**Figure 7.**
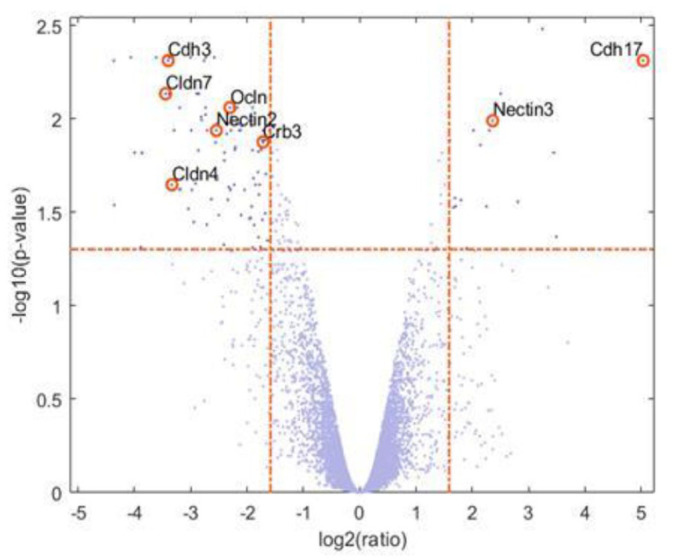
Overview of the differentially expressed genes in I12K9 versus I9K6 cells. Graph demonstrates a high similarity between both cell lines. Volcano plot of all 100 differently expressed genes where the p-value is plotted against the log2(ratio). Each dot represents one gene and the highlighted genes (right or left of the red dotted line) were significantly differentially expressed with a log fold change cut-off of 2. Mostly tight junction markers are significantly upregulated in I12K9 cells when set in relation to I9K6 cells.

As shown in studies regarding the transport capacity or the gene expression profile, the murine cell lines have demonstrated similarities, which were further investigated by microarray analyses. For this purpose, the murine cell lines I9K6 (transduction in 2D) and I12K9 (transduction in 3D) were set into relation, as different immortalization strategies were used for these lines. The transcriptomic data of I9K6 and I12K9 cells displayed a high similarity to each other (Figure 7) as visualized in a volcano plot. In total, 100 significantly differentially expressed genes based on 18,233 analyzed genes were identified in the I12K9 cells compared to I9K6 cells. The top upregulated genes in I12K9 cells are OCLN, CLDN4, CLDN7, Cdh3, Nectin2, and Crb3, while Nectin3 and Cdh17 are upregulated in I9K6 cells as shown in Figure 7. Therefore, genes that have an impact on barrier integrity were mainly influenced by the applied immortalization strategy.

### Identification of differentially expressed genes in the human cell line compared to Caco-2 cells

**Figure 8.**
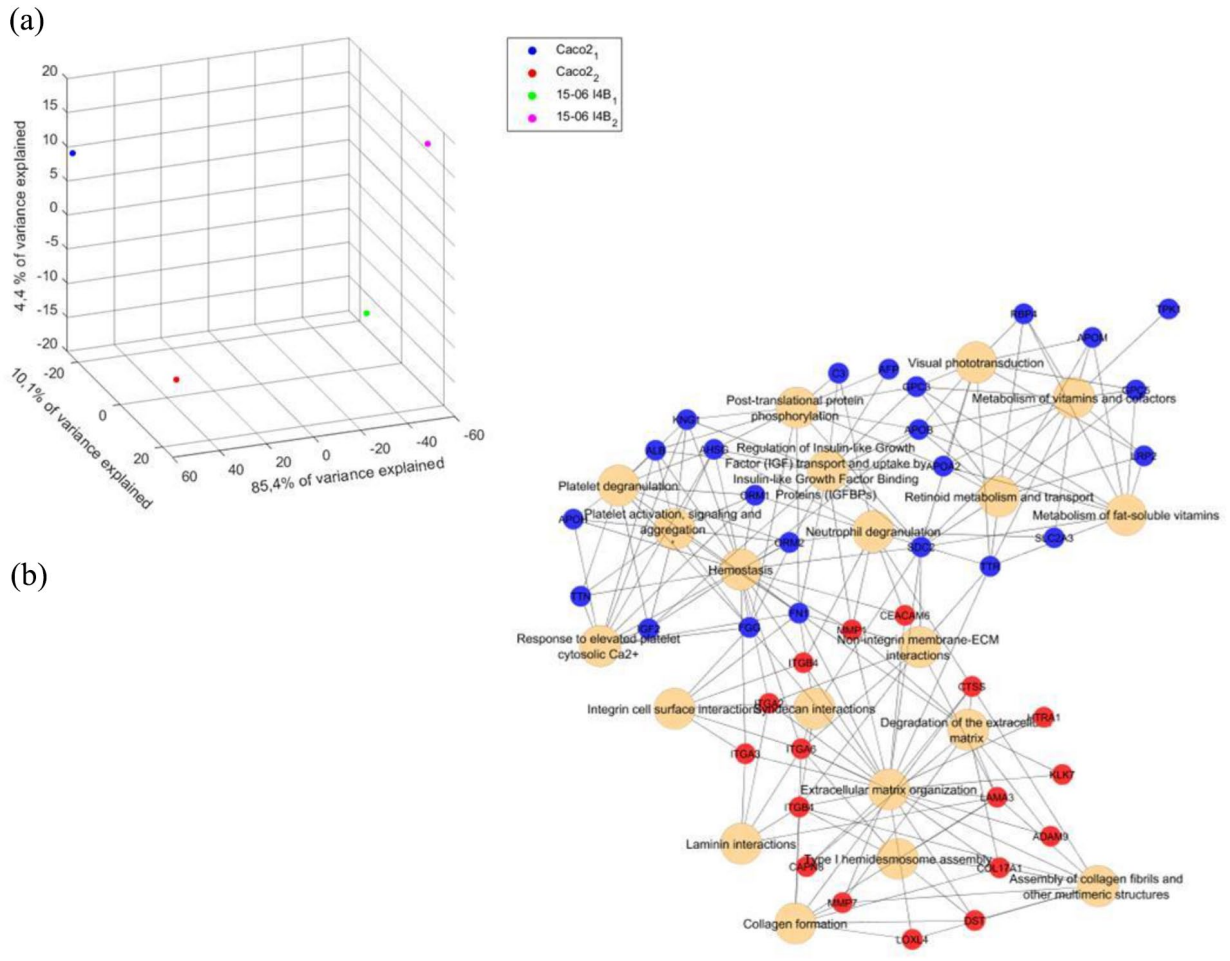
Principal component analysis (PCA) plot and pathway enrichment analyses shows differences in the gene expression profile of the human cell line compared to Caco-2 cells. PCA score 3D plot of different passages of the human cell line 15-06 I4B and the commonly known cell line Caco-2 (a). Network of significantly enriched (red) and diminished (blue) pathways together with the associated genes (b).

The generated human organoid-based cell line 15-06 I4B was likewise analyzed regarding its whole gene expression profile by microarray analysis. Therefore 15-06 I4B cells were compared to the commonly used Caco-2 cell line, both cultured for 14 or 21 days on Transwell®-inserts. The microarray analyses identified a total number of 400 differentially expressed genes between these two cell lines, which was further assessed through a multivariate analysis of gene expression data using principal component analysis (PCA). The PCA carried out on the total number of genes resulted in an 85.4% variance between the different cell lines and 10.1% variance within the analyzed passages for each cell line. Next, gene ontology (GO) and pathway enrichment analyses were performed for the 400 differently expressed genes to investigate their biological functions. In the GO analysis, all of the results were ranked and the top 15 results of the category biological process (BP) are shown in Supplemental Figure 3. The GO BP terms for genes significantly over-represented in 15-06 I4B cells were mainly associated with cell-adhesion (Supplemental Figure 3A). Transcriptional expressions of those genes participating in lipoprotein remodeling were significantly downregulated in our generated human cell line (Supplemental Figure 3B). The results of the pathway enrichment analyses were additionally ranked and the significantly enriched pathways are presented in Figure 8(b), which revealed enrichment in extracellular matrix organization-associated pathways in the established human cell line. In contrast, metabolic-associated pathways were decreased in 15-06 I4B cells in this network analysis (Figure 8(b)).

**Figure 9.**
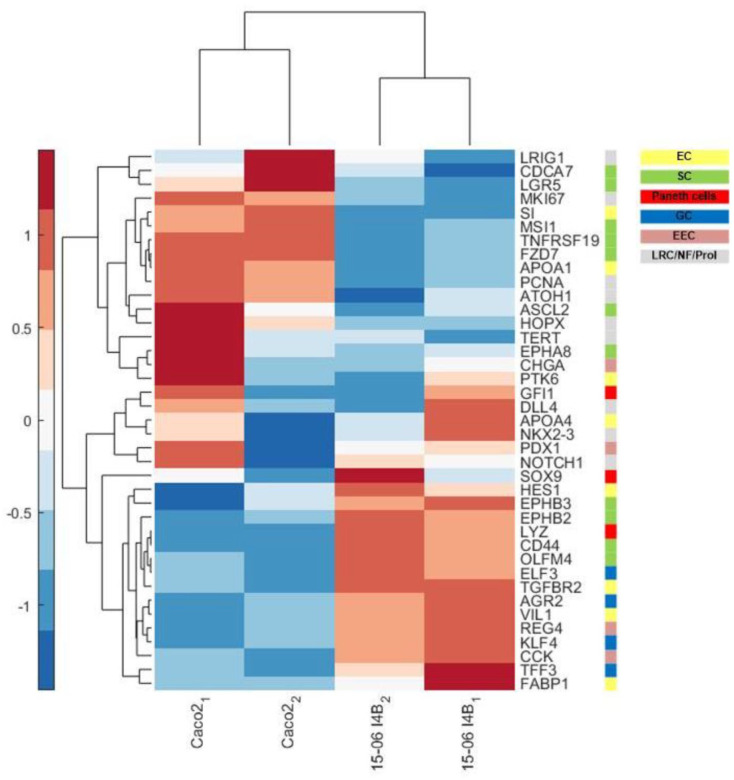
Cell-type specific transcript of the generated human cell line differs from Caco-2 cells. Cell-type specific transcripts for absorptive cells/enterocytes (Abs/EC), goblet cells (GC), enteroendocrine cells (EEC), Paneth cells (PC), and crypt base columnar and stem cells (CBC/SC). Heatmap shows genes described for the different cell types located in the intestinal epithelium, which were differently expressed in the generated human cell line compared to Caco-2 cells.

To further validate these differences of the generated human cell line to the commonly known ‘gold standard’ Caco-2, we examined the microarray data for the expression of well-known cell type-specific genes for absorptive cells/enterocytes (Abs/EC), goblet cells (GC), enteroendocrine cells (EEC), Paneth cells (PC), and crypt base columnar and stem cells (CBC/SC; Figure 9). The color bar in the clustergram indicates the z-score of the gene expression level resulting from the microarray analyses. Genes representative for the respective cell types are marked with different color codes as shown in Figure 9. Most of the cell-type specific markers described in the context of differentiated cells exhibited a higher expression in the human cell line, while most of the stem cell markers such as *LGR5* showed a higher expression in Caco-2 cells. As already shown in RT-qPCR data, the expression of *CHGA* as marker for EECs was rather low; however, other EEC-specific markers were identified for our human cell line (Figure 9). Moreover, only a small variation in the mapping between biological replicates was observed, suggestive of consistency between the two replicates marked as 1 and 2 for each cell line.

**Figure 10.**
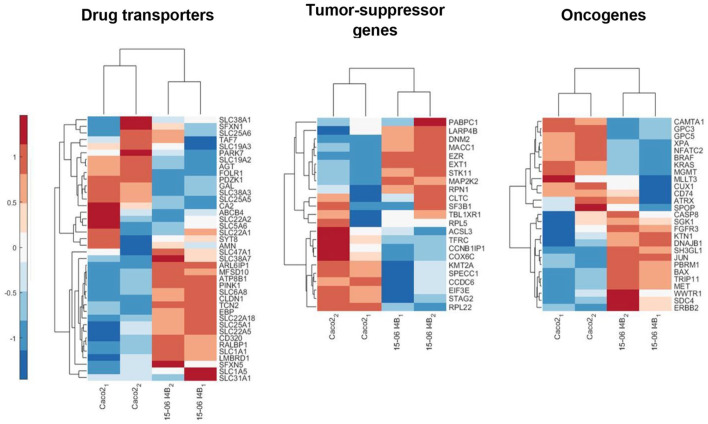
Heatmap of the generated human cell line differs from Caco-2 cells regarding the gene expression profile of specific epithelial transporters and tumor background. Heatmap shows the transcriptomic signature of specific epithelial drug transporters. Heatmap demonstrates the expression profile of oncogenes and tumor-suppressor genes. Each heatmap was generated with a sample size of 2 (different passages).

To exhibit the relevance of our models in the context of drug evaluation and efficacy tests, the expression of specific drug transporters was investigated. As shown in Figure 10, the general expression profile of the newly generated human cell line-based models differs from that of Caco-2 cell-based models. With the help of the human protein atlas, the corresponding SLC transporters were identified to be more colon-specific for the Caco-2 cells (e.g., *SLC22A1*, *SLC38A1*, and *SLC5A6*), while the 15-06 I4B cells showed a profile that more closely resembles that of the small intestine (e.g., *SLC6A8*, *SLC22A5*, and *SLC31A1*). In general, the SLC transporter genes with the highest difference in expression were found to be related to amino acid transporters, organic cation/anion/zwitterion transporters and mitochondrial carriers.^32^ For the other genes classified as drug transporters such as *TCN2*, *ARL6IP1*, or *EBP* being upregulated in the human cell line an assignment in the human protein atlas to the small intestine is also recognizable. However, genes like *PDZK1*, *GAL*, or *TAF7* upregulated in Caco-2 cells can be localized according to the protein atlas in colon tissue as well as in the small intestinal epithelium.

Furthermore, as our human cell line was generated by lentiviral transduction with different oncogenes, we selected out genes associated to colorectal cancer according to the publication of Shin et al.^33^ and grouped them likewise into oncogenes and tumor-suppressor genes. For both cell lines tested, expression of tumor-suppressor genes and oncogenes was observed but with different expression levels as shown in Figure 10. However, the tumor-suppressor genes as well as the oncogenes revealed a strong difference in the gene expression profile for Caco-2 cell-based models compared to our human cell line-based models, thereby indicating a clear distinction to the tumor background of the Caco-2 cells.

## Discussion and conclusion

Orally applied drugs have to overcome the intestinal barrier to enter the bloodstream and thereby reach their target site. Therefore, the bioavailability of drugs is strongly regulated by the absorption and transport mechanisms present in the small intestine. To mimic such processes in vitro and to determine the effectiveness and toxicity of newly developed drugs, predictive pre-clinical models are required. Tumor-derived Caco-2 cells cultured either in 2D or on artificial PET membranes resembling 3D-like cell culture technologies have advanced our knowledge of effective dosing, toxicity, or bioavailability of drugs.^9,10,25,34^ Nevertheless, high failure rates have been observed in drug discovery due to culture conditions and the tumor entity of these cells.^35^ To overcome such difficulties, primary organoid-based models were developed either as Transwell®-like models or organ-on-chip models.^14,16,36^ However, their complex handling, high costs, and low standardization do not allow application in pharmaceutical research so far.

Therefore intestinal in vitro models that meet criteria such as presenting a standardized system, being high-throughput qualified and revealing structural as well as functional characteristics comparable to the in vivo situation, all while being easy to handle under cost-efficient conditions, are urgently needed. To achieve this, an alternative cell source based on primary intestinal organoid cultures was established in our study by a lentiviral transduction strategy. Here, we used a combination of several immortalizing genes such as ID1, ID2, E7, Nanog, or Fos to induce immortality in dividing as well as non-dividing cells for the establishment of an intestinal organoid-based cell line. This immortalization strategy allows flexibility and reproducibility which further facilitates the process transfer to other donors, species, or other organ systems, as recently shown for human endothelial cells, murine embryonic intestinal epithelial cells, or alveolar lung cells.^20,22^ For instance, studies by Schwerk et al. showed the generation of murine intestinal epithelial cell lines with preserved primary cell-based properties to a particularly high degree, which were used in the context of cellular responses to type I and III interferon after an innate immune stimulus.^22^ Due to previous studies and an increased complexity of human organoid cultures, we first immortalized primary murine organoid cultures followed by human organoid cultures. In addition, genetic manipulation of 3D cultured cells is often inefficient as already demonstrated by Onuma et al.^37^ for murine primary intestinal cells. In order to improve the immortalization process, differentiated organoids achieved by the extension of culture time were genetically manipulated when cultured in 2D and 3D. Directly after immortalization several cell lines were picked and pre-screened; however, only three murine and one human cell line fulfilled the defined criteria such as a homogenous morphology or the presentation of a primary-like cell phenotype.

Our newly engineered murine and human cell lines representing the intestinal epithelium can be maintained in 2D similar to standard cell lines regarding freeze/thaw procedures and can be sub-cultured under cost-efficient conditions, as Matrigel® is no longer needed for cell expansion.^8^ In addition, successful immortalization and the preservation of certain in vivo-like characteristics was confirmed by proliferation assays.^14,38–40^ To further prove integration of immortalization genes and therefore indefinite growth, maintenance of a short telomere length and simultaneously high activity either by telomerase or alternative lengthening of telomeres (ALT) could be measured^40–42^ in future studies.

Next, cell lines were cultured in a 3D Matrigel® environment to identify, if cell aggregates would form and depict a typical organoid morphology. Similar to Caco-2 cells or other cell lines, they formed spheroid-like structures but no typical organoid structures, with separation into crypt-villus-domains, and showed a reduced proliferation capacity when cultured in 3D instead of 2D.^43–45^ However, cell aggregates formed by our newly generated cell lines exhibit an internal lumen similar to intestinal primary organoid cultures or Caco-2 cell-based spheroids. This feature seems to be specific only for some cell lines, as this was not demonstrated for HT-29 spheroids.^46^ For Caco-2 cell-based spheroids it is already known that this hollow structure is essential for the functional polarization through E-cadherin-dependent cell-cell adhesions.^46^ Due to this polarization, tight junction markers such as ZO-1 are identified on the apical inner side of the spheroids, similar to primary organoids as shown by IF staining.^47^ However, this fact could not be confirmed for our cell line-based spheroid-like structures as shown in IF analyses for VIL1. Probably spheroid formation based on single cells cultured in Matrigel® contributed to the incorporation of Matrigel® and the reversal of polarization. A specific reversal of Caco-2 spheroids was shown by Samy et al. when Matrigel® encapsulation occurs ^47^. A general change due to immortalization can be ruled out, as the cells showed a polarized epithelial cell layer on PET-based membranes. Taken together, the proliferative potential of our organoid-based cell lines is based on the immortalized differentiated cell populations and no functional stem cells are present anymore, as spheroid-like structures are more similar to Caco-2 spheroids instead of primary organoids.

The expression analysis of specific marker proteins representing the intestinal epithelium confirmed that cell lines with a differentiated cell phenotype were generated. Cell-type specific marker genes for enterocytes, goblet cells, and Paneth cells were identified.^2,14^ While, enteroendocrine cells identified by the marker gene *hCHGA/mChga* were not detected by gene expression analyses for our cell lines, microarray analyses showed an expression for other cell type-specific genes already described for enteroendocrine cells.^48^ The performed microarray analyses especially highlighted the differences between our cell line-based models and standard Caco-2 cell-based models, indicating a differentiated but intermediate cell type with characteristics similar to the in vivo situation. Mucus-producing goblet cells identified by the expression of TFF3 (Trefoil Factor 3).^49^ are of high importance in such models as the mucus layer provides an additional physical barrier and therefore affects transport mechanisms to a high degree.^50,51^ However, we cannot detect a fully mature epithelium for our generated cell lines, as changes in the expression level are caused by culture conditions. In addition, reduced expression levels are seen compared to primary organoids. These two observations have already been described for various cell lines.^52,53^ In this respect, further comparison of genes and proteins expressed in vivo would be of high interest.

The absorption of orally administered chemicals occurs primarily in the small intestine. Accordingly, a functional epithelial barrier is crucial in in vitro models to be used in pre-clinical drug discovery. In this context, our data demonstrate that the newly generated cell lines are capable of forming a functional barrier and therefore offer great potential for use as a pre-clinical study model. Our human cell line cultured on standard Transwell®-inserts develops a functional barrier after 14 days of culture, while our murine cell lines need 21 days until a confluent and functional epithelial cell layer is formed. Therefore, human cell line based models are comparable to primary organoid-based models even if the TEER value is four times as high.^14^ Compared to the native tissue, it is likewise four times higher.^14,54^ In general, our human cell line based models show substantially lower TEER values than Caco-2 cell-based models (~200–1000 Ω cm²), while murine cell line-based models display a similar TEER.^9,23,54^ However, the different cell culture protocols have a huge impact on the TEER value.^55,56^ With regard to the expression levels of various tight and adherence junction proteins, we observed a lack in the expression of CLDN5 in our human cell line, whereas other markers are uniformly expressed. This could be a sign for a tumor characteristic of this line,^57,58^ but this would contradict the microarray data. Therefore, after immortalization, the maintenance of the barrier integrity seems to be regulated by other claudin proteins as no reduced barrier integrity was observed in comparison to primary cell-based models.^14,59,60^

Ultrastructural analyses revealed further important characteristics for a functional barrier. Microvilli on the apical cell surface were shown to be comparable to primary organoids.^61,62^ These properties are strongly dependent on the scaffold material, coating, cell passage, and general culture conditions used. Therefore, improvements are still possible depending on the desired application. In addition, Mustata et al. observed likewise only a few and short microvilli for spheroids composed of mainly SCs and TACs, which could be a further indication that mostly TACs were immortalized.^61^

Transport mechanisms such as paracellular, transcellular, or efflux transport were investigated by representative reference substances and additionally demonstrated the functional performance of our primary-based cell line models.^25,63^ For comparison, Caco-2 cell-based models were implemented in our study to demonstrate the benefits of our newly generated cell lines. With regard to transcellular transport, our murine cell line-based models showed a higher transport rate than Caco-2 cell-based models or human cell line-based models, while the highest efflux rate was seen in our human cell-based models.^9,23,25^ Further, they show to some extent a comparability to the in vivo situation.^64,65^ Our microarray data further demonstrate that transporters/enzymes located in the apical cell membrane are differentially expressed in our models compared to Caco-2 models, indicating a better predictiveness in bioavailability studies. However, functionality of these transporters/enzymes should be verified in additional transport studies. In addition, these data revealed for our human cell line a profile that more closely resembles that of the small intestine as SLC transporters such as *SLC22A5*, *SLC1A1*, or *SLC6A8*, mainly localized in the small intestinal epithelium, are upregulated.^32,33,66^ In contrast, Caco-2 cell-based models demonstrated in our study a mixed expression profile for genes localized in the small intestinal epithelium as well as in the colon tissue.^32,33,66,67^ This immature state of Caco-2 cells is also reflected in cell type-specific gene analysis, as we observed a higher expression level for stem cell or progenitor-associated genes compared to our human cell line. With regard to the expressed genes representative for the different cell-types localized in the small intestinal epithelium, our human cell line is also more comparable to the in vivo situation than Caco-2 cell-based models.

In addition, we investigated in our microarray analysis oncogenes and tumor-suppressor genes, which were already described in the context of Caco-2 cells.^33^ Oncogenes such as *GPC3*, *MGMT*, *KRAS*, or *CAMTA1* are highly expressed in Caco-2 cell-based models as shown by Shin et al.,^33^ showed in our study a likewise high expression compared to our human cell line. However, genes expressed to a lower extend in Caco-2 cell-based models such as *SGK1*, *KTN1*, or *MET*^33^ revealed for our human cell line-based models a higher expression. A similar gene expression profile emerges for the tumor-suppressor genes in our study. The expression profile of Caco-2 cell-based models is similar to literature,^33^ while our human cell line shows the opposite gene expression profile to Caco-2 cells. Therefore, the microarray data further support our findings, that our generated human cell line is more likely to have an epithelial phenotype than Caco-2 cells.

Taken together, our study demonstrated that the cellular diversity of primary intestinal organoids is sustained in the newly generated cell lines of human and murine origin after immortalization, while establishing a cell culture population which is easy to handle under cost-efficient conditions and thus fulfilling the requirements for pre-clinical research purposes. Most notably, we demonstrate that the newly generated cell lines form a functional barrier to study cellular responses and drug efficacy under simplified conditions with high significance.

## Supplemental Material

sj-docx-1-tej-10.1177_20417314241228949 – Supplemental material for Enhancing pre-clinical research with simplified intestinal cell line models Supplemental material, sj-docx-1-tej-10.1177_20417314241228949 for Enhancing pre-clinical research with simplified intestinal cell line models by Christina Fey, Theresa Truschel, Kristina Nehlsen, Spyridon Damigos, Julia Horstmann, Theresia Stradal, Tobias May, Marco Metzger and Daniela Zdzieblo in Journal of Tissue Engineering
