# Supplementary material for: Enhancing pre-clinical research with simplified intestinal cell line models
Source: J Tissue Eng. 2024 Mar 4;15:20417314241228949. doi: 10.1177/20417314241228949 (PMC10916479; doi:10.1177/20417314241228949)
Supplement: sj-docx-1-tej-10.1177_20417314241228949 – Supplemental material for Enhancing pre-clinical research with simplified intestinal cell line models [file sj-docx-1-tej-10.1177_20417314241228949.docx]

**Research Paper**

**Enhancing Pre-Clinical Research with Simplified Intestinal Cell Line Models**

*C. Fey^1^, T. Truschel^2^, K. Nehlsen^2^, S. Damigos^4^, J. Horstmann^3^, T. Stradal^3^, T. May^2^, M. Metzger^1,4^, D. Zdzieblo^4,5^*

^1^ Translational Center for Regenerative Therapies (TLZ-RT) Würzburg, Branch of the Fraunhofer Institute for Silicate Research (ISC), Würzburg, Germany.

^2^ InSCREENeX GmbH, Braunschweig, Germany.

^3^ Helmholtz Centre for Infection Research, Braunschweig, Germany.

^4^ Department of Tissue Engineering and Regenerative Medicine (TERM), University Hospital Würzburg, Würzburg, Germany.

^5^ Project Center for Stem Cell Process Engineering (PZ-SPT), Fraunhofer Institute for Silicate Research (ISC), Würzburg, Germany.

**Supplementary information**

Supplementary Table 1: Murine and human donor tissue biopsies. m/#: murine; h/: human; f: female; m: male; w: weeks; y: years.

| **Species/Number** | **Segment** | **Gender** | **Age** |
| --- | --- | --- | --- |
| m/#110 | jejunum | m | 15 w |
| m/#9 | jejunum | m | 16 w |
| m/#114 | jejunum | f | 27 w |
| m/#123 | jejunum | f | 23 w |
| h/14-08 | duodenum | f | 43 y |
| h/15-01 | duodenum | m | 52 y |
| h/15-05 | duodenum | m | 66 y |
| h/15-06 | duodenum | f | 30 y |

Supplementary Table 2: Integration of library genes in murine and human cell lines from primary organoid-derived intestinal epithelial cells. Murine and human cell lines were analyzed for the integrated library genes by PCR. Grey: gene integrated; star: gene only used for human cell line.

| **cell type** | | **I9K6** | **I9K8** | **I12K9** | **15-06 I4B** |
| --- | --- | --- | --- | --- | --- |
| **donor** | | m/#123 | m/#123 | m/#123 | h/15-06 |
| **genes** | *E6** |  |  |  |  |
|  | *E7* |  |  |  |  |
|  | *Nanog* |  |  |  |  |
|  | *cMyc* |  |  |  |  |
|  | *Id1* |  |  |  |  |
|  | *Id2* |  |  |  |  |
|  | *Id3* |  |  |  |  |
|  | *Fos* |  |  |  |  |
|  | *TaG* |  |  |  |  |
|  | *Core* |  |  |  |  |
|  | *Rex* |  |  |  |  |
|  | *Bmi1** |  |  |  |  |

Supplementary Table 3: Medium and supplements used for intestinal epithelial cell cultures.

| **Reagent name** | **Supplier** | **Cat No.** | **Final conc** |
| --- | --- | --- | --- |
| Advanced DMEM/F12 | Gibco | 12634-028 |  |
| GlutaMAX-I | Gibco | 35050-061 (100x) | 1x (v/v) |
| HEPES | Sigma Aldrich | H3662-1M | 10 mM |
| Anti-Anti | Gibco | 15240-062 (100x) | 1x (v/v) |
| N-2 supplement | Gibco | 17502-048 (100x) | 1% (v/v) |
| B-27 supplement (without Vitamin A) | Gibco | 12587-010 (50x) | 0.5% (v/v) |
| *N*-Acetylcysteine | Sigma Aldrich | A9165 | 1 mM |
| rec mNoggin | Peprotech | 250-38 | 100 ng/mL |
| rec hEGF | Peprotech | AF-100-15 | 50 ng/mL |
| rec hR-Spondin | Peprotech | 120-38 | 500 ng/mL |
| Y-27632 | Tocris Bioscience | 1254 | 10 µM |
| A83-01 | Tocris Bioscience | 2939 | 500 nM |
| SB202190 | Sigma Aldrich | S7067 | 10 µM |
| Nicotinamide | Sigma Aldrich | N0636 | 10 mM |
| [Leu15]-Gastrin I | Sigma Aldrich | G9145 | 10 mM |
| LY2157299 | Axon MedChem | 1491 | 500 mM |
| JAG-1 | AnaSpec Inc. | AS-61298 | 10 µM |
| CHIR99021 | Biomol | Cay13122 | 3 µM |
| Valproic acid | Sigma Aldrich | P4543 | 1 mM |
| FCS | Bio&Cell | LE.0.500 | 10% |
| DMEM | Gibco | 32430100 |  |
| G-418 | Invivogen | ant-gn-1 |  |
| FCS | Bio&Cell | LE.0.500 | 10% (v/v) |
| Sodium pyruvate | Gibco | 11360070 | 1% |

| **Crypt medium** | **Wnt3a-conditioned medium** | **mSM** | **hSM** |
| --- | --- | --- | --- |
| Advanced DMEM/F12 | DMEM | Crypt medium (50%) | Crypt medium (50%) |
| GlutaMAX-I | G-418 | Wnt3a-conditioned medium (50%) | Wnt3a-conditioned medium (50%) |
| HEPES | FCS | rec mNoggin | rec mNoggin |
| Anti-Anti | Sodium pyruvate | rec hEGF | rec hEGF |
| N-2 supplement |  | rec hR-Spondin | rec hR-Spondin |
| B-27 supplement (without Vitamin A) |  | Y-27632 | Y-27632 |
| *N*-Acetylcysteine |  | CHIR99021 | A83-01 |
|  |  | Valproic acid | SB202190 |
|  |  |  | Nicotinamide |
|  |  |  | [Leu15]-Gastrin I |
|  |  |  | LY2157299 |
|  |  |  | JAG-1 (single cells) |

Supplementary Table 4: Medium composition for murine and human spheroids. mSM: murine spheroid maintenance medium; hSM: human spheroid maintenance medium; Y-27632 supplemented only for 2 days after splitting in mSM and hSM medium.

| **mCL** | **hCL** |
| --- | --- |
| Crypt medium | Crypt medium (50%) |
| rec mNoggin | Wnt3a-conditioned medium (50%) |
| rec hEGF | rec mNoggin |
| Y-27632 | rec hEGF |
| FCS | rec hR-Spondin |
|  | Y-27632 |
|  | A83-01 |
|  | SB202190 |
|  | Nicotinamide |
|  | [Leu15]-Gastrin I |
|  | LY2157299 |

Supplementary Table 5: Medium composition for murine and human cell line. mCL: murine cell line medium; hCL: human cell line medium.

Supplementary Table 6: Primary and secondary antibodies for histological characterization.

| **Antibody** | **Host** | **Dilution** | **Supplier (order number)** |
| --- | --- | --- | --- |
| **Primary antibodies** | | | |
| anti-E-Cadherin | Mouse | 1:100 | BD Biosciences (610181) |
| anti-Ki-67 | Rabbit | 1:100 | Abcam (ab15580) |
| anti-Mucin 2 | Rabbit | 1:100 | Abcam (ab272692) |
| anti-Occludin | Mouse | 1:100 | Invitrogen (33-1500) |
| anti-Villin 1 | Goat | 1:100 | Santa Cruz (sc-7672) |
| anti-Zonula occludens-1 | Rabbit | 1:100 | Proteintech (21773-1-AP) |
| **Secondary antibodies** | | | |
| anti-mouse Alexa 555 | Donkey | 1:400 | Invitrogen (A-31570) |
| anti-mouse Alexa 647 | Donkey | 1:400 | Invitrogen (A-31571) |
| anti-rabbit Alexa 555 | Donkey | 1:400 | Invitrogen (A-31572) |
| anti-goat Alexa 647 | Donkey | 1:400 | Invitrogen (A-21447) |

Supplementary Table 7: Polymerase chain reaction primer pairs.

| **Name** | **5’ to 3’ Sequence** | **Source** |
| --- | --- | --- |
| *mRpl15* | 5’-CTGACCCTGGATGTCTTGGTGC-3’  5’-CCAAGCAGCCACTTCAGTGAACC-3’ | NM_025586.3  (housekeeping gene) |
| *mRps29* | 5’-GTCTGATCCGCAAATACGGG-3‘  5’-AGCCTATGTCCTTCGCGTACT-3‘ | NM_009093  (housekeeping gene) |
| *mLgr5* | 5’-GGGAAGCGTTCACGGGCCTTC-3’  5’-GGTTGGCATCTAGGCGCAGGG-3’ | NM_010195.2 |
| *mMuc2* | 5’-GCTGCATTTGCCGGAACGGG-3’  5’-GGCAGCTAGTGGGACGGGGT-3’ | NM_023566.3 |
| *mLyz* | 5’-CCTGACTCTGGGACTCCTCCTGC-3’  5’-CCACGGTTGTAGTTTGTAGCTCGT-3’ | NM_017372.3 |
| *mVil1* | 5’-GCAGCATTACCTGCTCTACGTT-3’  5’-GCTTGATAAGCTGATGCTGTAATTT-3’ | NM_007127.2 |
| *mChga* | 5’-AGAATTTACTGAAGAAGCTCCAAG-3’  5’-TCCTCTCTTTTCTCCATAACATCC-3’ | NM_001275.3 |
| *hHPRT1* | 5’-TGACCTTGATTTATTTTGCATACC-3’  3’-CGAGCAAGACGTTCAGTCCT-3’ | NM_000194.2  (housekeeping gene) |
| *hEF1α* | 5‘-AGGTGATTATCCTGAACCATCC-3‘  5‘-AAAGGTGGATAGTCTGAGAAGC-3‘ | [NM_001402.5](http://www.ncbi.nlm.nih.gov/entrez/viewer.fcgi?db=nucleotide&id=83367078)  (housekeeping gene) |
| *hMUC2* | 5’-AGGATCTGAAGAAGTGTGTCACTG-3’  5’-TAATGGAACAGATGTTGAAGTGCT-3’ | NM_002457.3 |
| *hVIL1* | 5‘-GCAGCATTACCTGCTCTACGTT-3‘  5‘-GCTTGATAAGCTGATGCTGTAATTT-3‘ | [NM_007127.2](http://www.ncbi.nlm.nih.gov/entrez/viewer.fcgi?db=nucleotide&id=194394236) |
| *hCHGA* | 5‘-AGAATTTACTGAAGAAGCTCCAAG-3‘  5‘-TCCTCTCTTTTCTCCATAACATCC-3‘ | NM_001275.3 |
| *hLYZ* | 5‘-CCGCTACTGGTGTAATGATGG-3‘  5‘-CATCAGCGATGTTATCTTGCAG-3‘ | [NM_000239.2](http://www.ncbi.nlm.nih.gov/entrez/viewer.fcgi?db=nucleotide&id=169790843) |
| *hLGR5* | 5‘-TCACCTTCCCCAGGCCCCTTC-3‘  5‘-TGTTCACTGCTGCGATGACCCC-3‘ | NM_003667.3 |


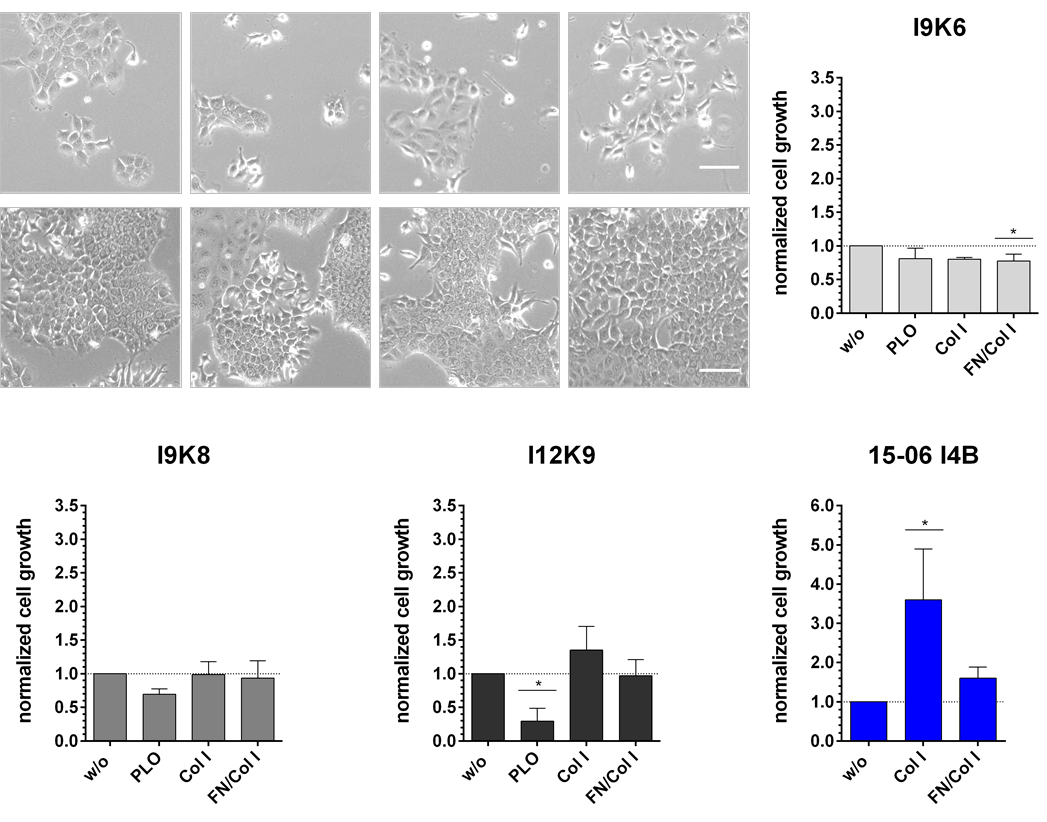


**A**

**B**

**w/o**

**PLO**

**Col I**

**FN/Col I**

**E**

**Cell morphology I9K6**

**C**

**D**

**d2**

**d4**

Supplementary Figure 1: Cell growth is influenced minimally when cultured under reduced conditions. Murine cell clones (I9K6, I9K8 and I12K9) were cultured on uncoated (w/o), PLO, Col I or FN/Col I pre-coated surfaces for 4-5 days, while the human cell clone (15-06 I4B) was cultured on uncoated (w/o), Col I and FN/Col I pre-coated surfaces for 7 days. Cell morphology (A, representative for I9K6) as well as cell growth were monitored (B-E). Graphs demonstrate normalized cell growth. The increase in cell numbers was normalized to the cell growth in uncoated cultures. Scale bar = 100 µm. Statistical analyses were carried out by one-way ANOVA. **p<0.05*; n = 3.


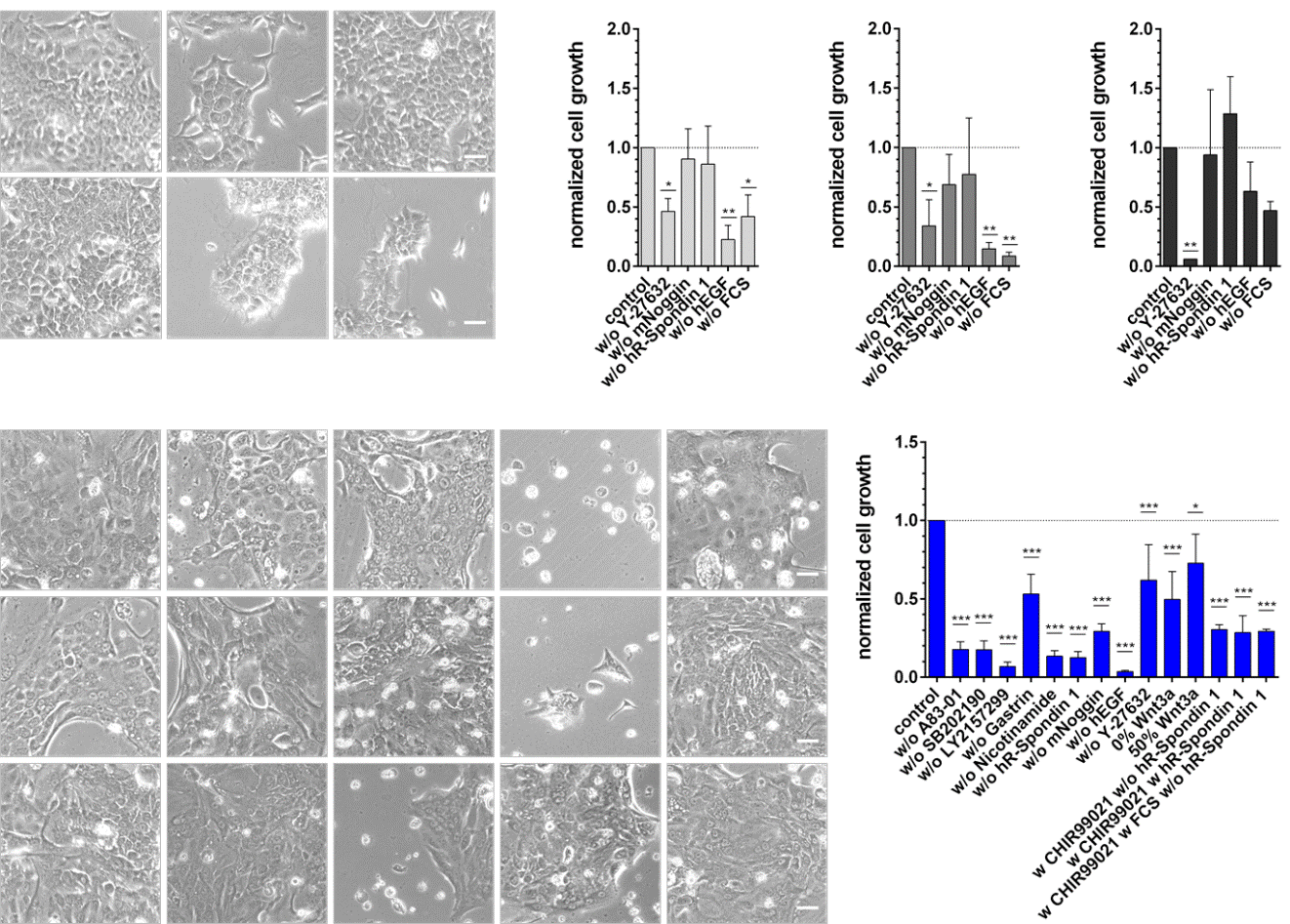


**A**

**B**

**C**

**D**

**E**

**F**

**control**

**w/o Nicotinamide**

**0% Wnt3a**

**w/o A83-01**

**w/o hR-Spondin1**

**50% Wnt3a**

**w/o SB202190**

**w/o Noggin**

**w CHIR99021**

**w/o hR-Spondin1**

**w/o LY2157299**

**w/o EGF**

**w CHIR99021**

**w hR-Spondin1**

**w/o Gastrin**

**w/o Y-27632**

**w CHIR99021**

**w FCS**

**w/o hR-Spondin1**

**control**

**w/o Y-27632**

**w/o Noggin**

**w/o hR-Spondin1**

**w/o hEGF**

**w/o FCS**

Supplementary Figure 2: Testing culture conditions in comparison to the standard medium for murine and human organoids. Cell clones were cultured under control conditions (standard medium for murine and human organoid cultures) and without the essential growth factors for primary spheroid/organoid cultures (A, E) (Sato et al. 2011; Sato et al. 2009). For murine cell clone cultures (A = I9K6, B = I9K8, C = I12K9, D = 15-06 I4B; A-D) 10% FCS was directly added after lentiviral transduction, as it is commonly used for culture of cell lines. For human cell clones (E-F) different combinations of growth factors influencing the Wnt pathway were tested. Cell morphology (A = representative for I9K6, E) and cell growth (B-D, F) were monitored for murine cell clones on d4-5 and for the human cell clone on d7. Normalized cell growth analysis demonstrated that all factors used for human spheroid cultures were essential for the human cell clone (F), while murine cell clones can be cultured under reduced culture conditions (B-D). Scale bar = 50 µm. Significance was calculated by one-way ANOVA. **p<0.05*, ***<0.01*, ****p<0.001*; n = 3.


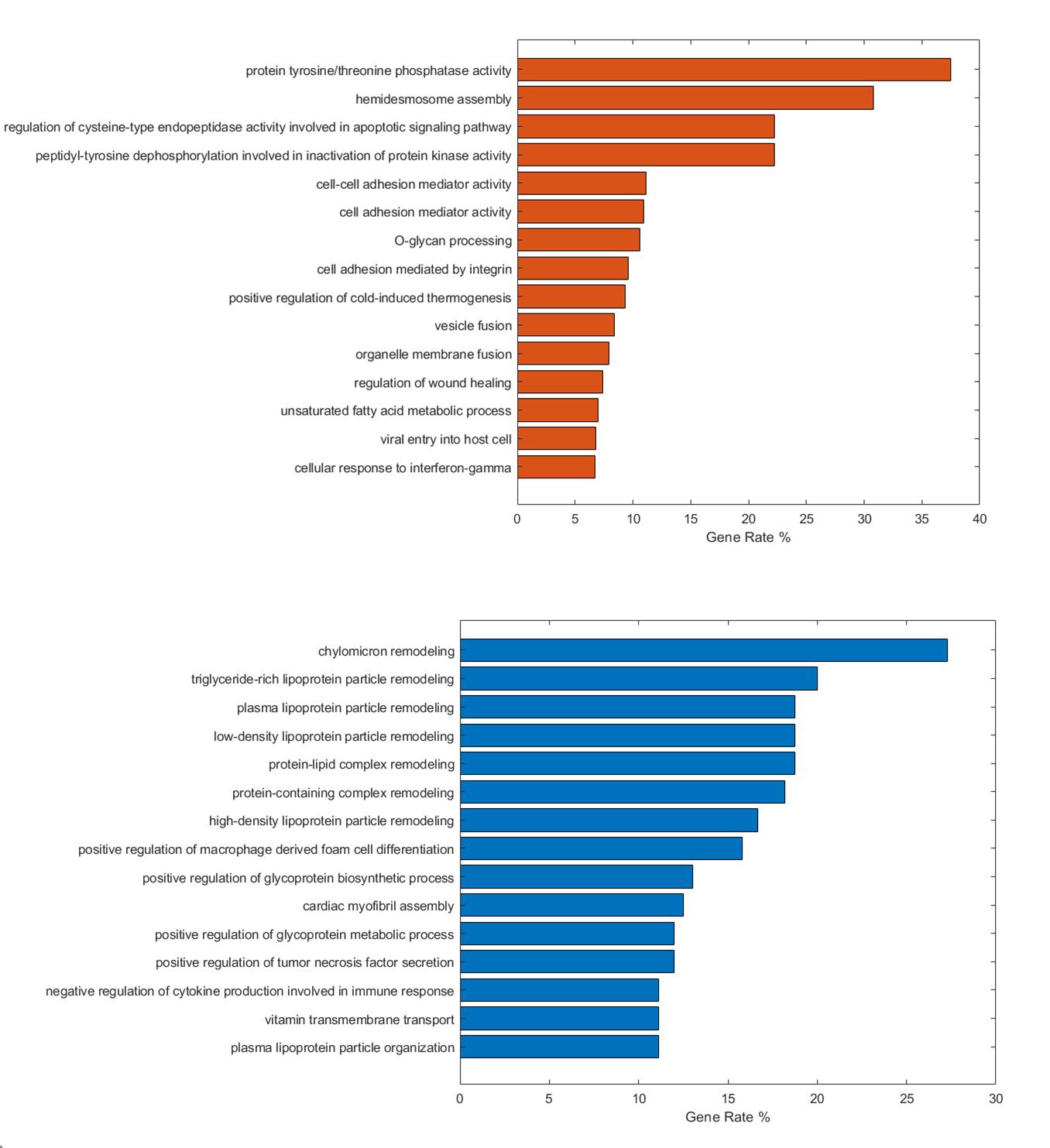


**B**

**A**

Supplementary Figure 3: Biological processes changed in human cell line compared to Caco-2 cells. Biological processes upregulated (A) and downregulated (B) in 15-06 I4B cell-based models.

References

Sato, Toshiro; Stange, Daniel E.; Ferrante, Marc; Vries, Robert G J; van Es, Johan H; Van den Brink, Stieneke et al. (2011): Long-term expansion of epithelial organoids from human colon, adenoma, adenocarcinoma, and Barrett's epithelium. In: *Gastroenterology* 141 (5), S. 1762–1772. DOI: 10.1053/j.gastro.2011.07.050.

Sato, Toshiro; Vries, Robert G.; Snippert, Hugo J.; van de Wetering, Marc; Barker, Nick; Stange, Daniel E. et al. (2009): Single Lgr5 stem cells build crypt-villus structures in vitro without a mesenchymal niche. In: *Nature* 459 (7244), S. 262–265. DOI: 10.1038/nature07935.
